# Supplementary material for: Severe Immune-Related Adverse Events in Patients Treated with Nivolumab for Metastatic Renal Cell Carcinoma Are Associated with PDCD1 Polymorphism
Source: Genes (Basel). 2022 Jul 5;13(7):1204. doi: 10.3390/genes13071204 (PMC9324515; doi:10.3390/genes13071204)
Supplement: Supplementary file 1 [file genes-13-01204-s001.zip › genes-1754433-supplementary.pdf]

Supplementary Table S1. Primers and PCR conditions for SNP typing of polymorphic variants of *PDCD1* gene

Gene	SNP	RS	primer sequences	Restriction enzyme	PCR product (bp)	RFLP band pattern (bp)
PDCD1	*PD-1.5 C>T*	rs2227981	5'-GTGCCTGTGTTCTCTGTGGA-3'<br>5'-CCAAGAGCAGTGTCCATCCT-3'	8 μL<br>Pvu II, 5U	212	C: 212<br>T: 156+56
	PD-1.3 G>A	rs11568821	5'-GCAGCAACCTCAATCCCTAA-3'<br>5'-AAATGTCCCTGGCATTCTTG-3'	8μL<br>Pst Ⅰ, 5U	222	G: 333<br>A: 51+282
	PD-1.6 G>A	rs10204525	5'- CCTCACACCACTCGGGAGA-3'<br>5'-AGTGGGGGTGCAGTGTGT-3'	8μL<br>Nla III, 5U	301	G: 164+137<br>A:301

SNP, single nucleotide polymorphism; *PDCD1, Programmed cell death protein 1*; RS, reference single nucleotide polymorphism identification number; RFLP, restriction fragment length polymorphism

Supplementary Table S2. Prognostic values of clinical variables and *PDCD1* polymorphism for predicting grade 2 or more irAE and multiple irAEs in 106 patients with metastatic renal cell carcinoma analyzed by using multivaluable logistic regression models

Factor	Risk category	At least one irAE ≥ G2			Multiple irAEs		
		OR	95% CI	p	OR	95% CI	p
Regimen	Nivo + Ipi				1.279	0.965 – 13.333	0.072
Clinical stage	3≦	2.183	0.645 – 7.392	0.210			
Nephrectomy	yes				0.800	0.223 – 2.866	0.731
PDCD1 SNP	*PD-1.6 G allele*	**3.712**	**1.591 – 8.658**	**0.002**	**3.047**	**1.151 – 8.065**	**0.025**

PDCD1, Programmed cell death protein 1; SNP, single nucleotide polymorphism; irAE, immune-related adverse event; G, grade; OR, odds ratio; CI, confidence interval
